# Supplementary material for: Explaining the Release Mechanism of Ritonavir/PVPVA Amorphous Solid Dispersions
Source: Pharmaceutics. 2022 Sep 8;14(9):1904. doi: 10.3390/pharmaceutics14091904 (PMC9505701; doi:10.3390/pharmaceutics14091904)
Supplement: Supplementary file 1 [file pharmaceutics-14-01904-s001.zip › pharmaceutics-1848517-supplementary.pdf]

pharmaceutics

Supplementary Material

Explaining the Release Mechanism of Ritonavir/PVPVA Amorphous Solid Dispersions

Adrian Krummnow [1,2], Andreas Danzer [1], Kristin Voges [2], Stefanie Dohrn [2], Samuel O. Kyeremateng [2,*], Matthias Degenhardt [2] and Gabriele Sadowski [1,*]

[1] Laboratory of Thermodynamics, Department of Biochemical and Chemical Engineering, TU Dortmund University, Emil-Figge-Str. 70, D-44227 Dortmund, Germany
[2] AbbVie Deutschland GmbH & Co. KG, Global Pharmaceutical R&D, Knollstraße, D-67061 Ludwigshafen am Rhein, Germany
* Correspondence: gabriele.sadowski@tu-dortmund.de (G.S.); samuel.kyeremateng@abbvie.com (S.O.K.); Tel.: +49-231-755-2635 (G.S.); +49-621-589-4940 (S.O.K.)

Citation: Krummnow, A.; Danzer, A.; Voges, K.; Dohrn, S.; Kyeremateng, S.O.; Degenhardt, M.; Sadowski, G. Explaining the Release Mechanism of Ritonavir/PVPVA Amorphous Solid Dispersions. *Pharmaceutics* **2022**, *14*, 1904. https://doi.org/10.3390/pharmaceutics14091904

Academic Editor: Antonello A. Barresi

Received: 19 July 2022
Accepted: 1 September 2022
Published: 8 September 2022

Publisher's Note: MDPI stays neutral with regard to jurisdictional claims in published maps and institutional affiliations.

Copyright: © 2022 by the authors. Submitted for possible open access publication under the terms and conditions of the Creative Commons Attribution (CC BY) license (https://creativecommons.org/licenses/by/4.0/).

S1 Phase diagram of PVPVA/water

Figure S1 shows the binary phase diagram and the glass-transition temperatures of PVPVA/water predicted with the parameters from Tables 1 to 4. At temperatures below 0 °C, water crystallizes from the PVPVA/water mixture in the S (solid) + L (liquid) region. The freezing-point depression of water with increasing PVPVA concentration corresponds to the solubility line of water in the water/PVPVA mixtures and was examined via mDSC measurements in this work. As to be seen in Figure S1, the experimental data are in very good agreement with the PC-SAFT prediction.

Between 0 °C and about 70 °C, PVPVA and water were predicted to form a single-phase liquid (L) over the whole concentration range. At higher temperatures, aqueous PVPVA solutions were predicted to show a miscibility gap (L1 + L2). This prediction perfectly matches with the experimental cloud-point temperature of 81 °C for an aqueous PVPVA solution (w_{PVPVA} = 0.01) obtained from literature [1] (Figure S1). The lower critical solution temperature (LCST) of approximately 73 °C measured in this work agrees very well with the reported value of 69.7 °C from Ritters et al. [2] as well as with the PC-SAFT prediction. At even higher temperatures, the PVPVA/water system reaches its boiling-point temperature and a vapor phase evolves (L + V) as predicted by PC-SAFT and also illustrated in Figure S1. It is worth noting that using one and the same parameter sets from Tables 3 and 4, PC-SAFT is able to predict the different kinds of phase equilibria in perfect agreement with experimental data.

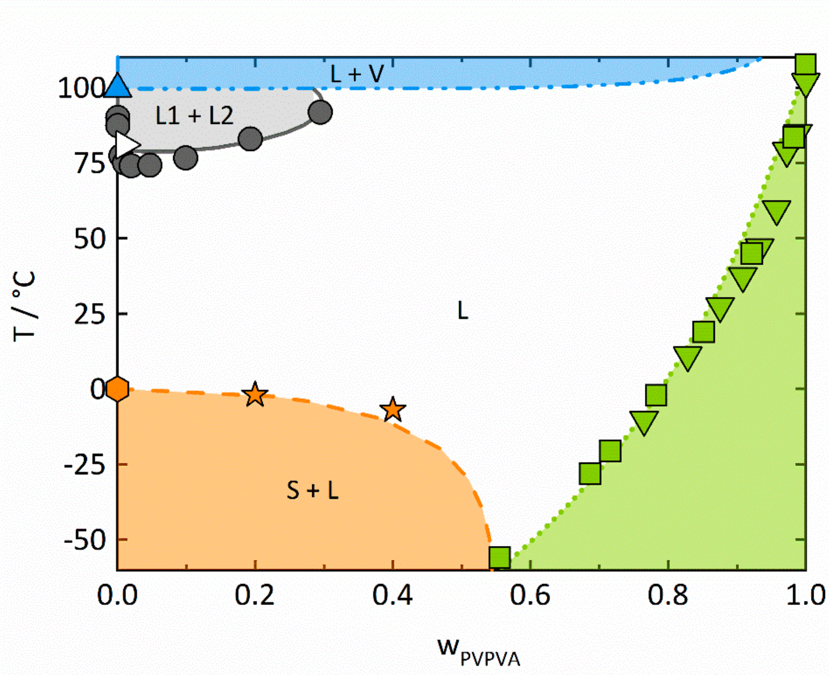

Figure S1. Binary phase diagram of PVPVA/water at 0.1 MPa with the solid-liquid equilibrium area S + L enclosed by the solubility line (orange dashed line), the miscibility gap L1 + L2 (gray solid line), and the vapor-liquid equilibrium L + V (blue dash-dotted line) calculated by PC-SAFT. The glass-transition temperature was calculated using the Kwei equation (green dotted line). Orange stars denote melting-point measurements via mDSC from this work. The orange hexagon is the melting point of water. Gray circles represent cloud-point measurements from this work and the white triangle to the right denotes a data point from Xi et al. [1]. The blue triangle upwards is the boiling temperature of water. Green squares denote glass-transition measurements from this work and green triangles downwards those from Taylor et al. [3].

S2 Phase diagram of RIT/water

Fig S2 shows the binary phase diagram and the glass-transition temperatures of RIT/water. At temperatures between 0 °C and 100 °C, RIT/water mixtures tend to phase-separate over almost the entire concentration range resulting in two coexisting liquid (amorphous) phases L1 + L2. The PC-SAFT calculations for the concentrations of the two coexisting phases are in excellent agreement with the experimental findings [4].

Amorphous RIT stored at 25 °C and 94% RH absorbs water leading to a RIT/water mixture with w_{water} = 0.046 as obtained in this work. As this composition lies within the calculated and experimentally determined miscibility gap, amorphous RIT stored at these conditions will form two phases.

The determined glass-transition temperatures were accurately modeled via the Kwei equation (ARD = 0.66%) and coincide with results from mDSC experiments by Sun et al. [5].

At temperatures below 125 °C, RIT crystallizes from the RIT/water mixture in the S (solid) + L (liquid). Solubilities calculated using PC-SAFT and measured via HPLC by Ilevbare et al. [4] are in good agreement. Above 100 °C, water starts to evaporate (L + V region). High RIT concentrations result in a remarkable boiling-point elevation of water as predicted by PC-SAFT.

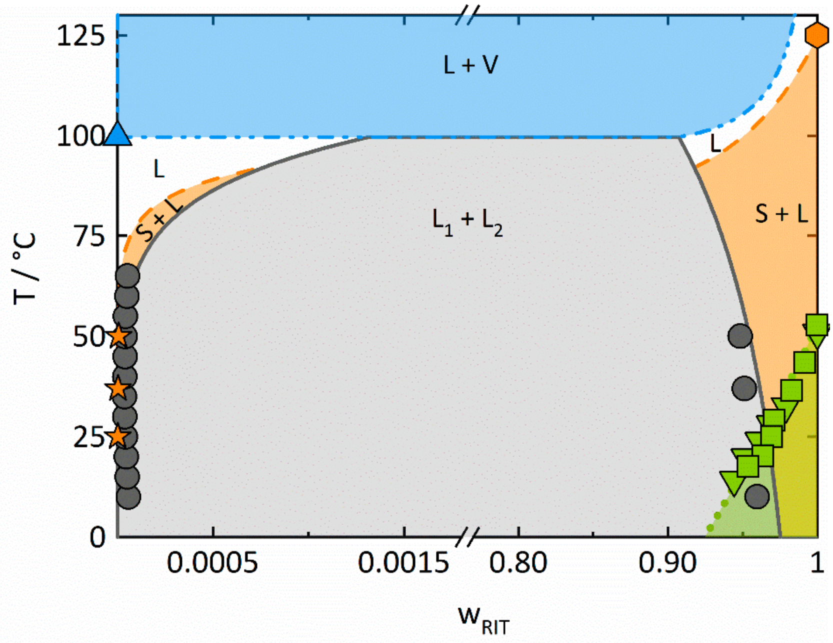

Figure S2. Binary phase diagram of RIT/water at 0.1 MPa with the solid-liquid equilibrium area S+L enclosed by the solubility line (orange dashed line), the miscibility gap L1 + L2 (gray solid line), and the vapor-liquid equilibrium L + V (blue dash-dotted line) calculated by PC-SAFT. The green dotted line is the glass-transition temperature according to the Kwei equation. Orange stars represent solubility measurements of Ilevbare et al. [4] and the orange hexagon of Dohrn et al. [6]. Gray circles denote liquid-liquid equilibrium measurements from Ilevbare et al. [4]. The blue triangle upwards is the boiling temperature of water. Green squares are glass-transition measurements from this work and green triangles downwards of Sun et al. [5].

Again, all presented phase diagrams were calculated based on the pure-component parameters and interaction parameters from Tables 4 and 5. These parameters apply regardless of the type of phase equilibria being considered.

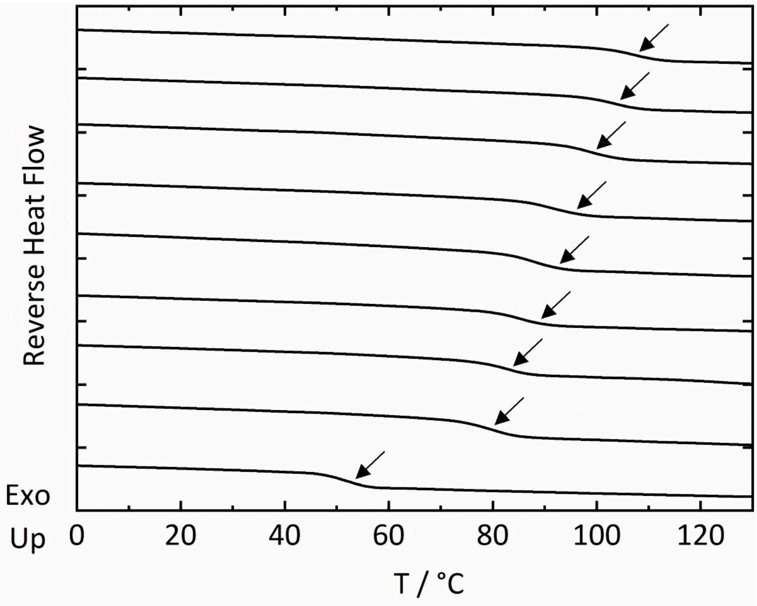

Figure S3. mDSC scans of dry RIT/PVPVA ASDs before dissolution for DLs of 0wt%, 10wt%, 20wt%, 25wt%, 30wt%, 35wt%, 40wt%, and 100wt% from top to bottom. Arrows indicate glass-transition temperatures.

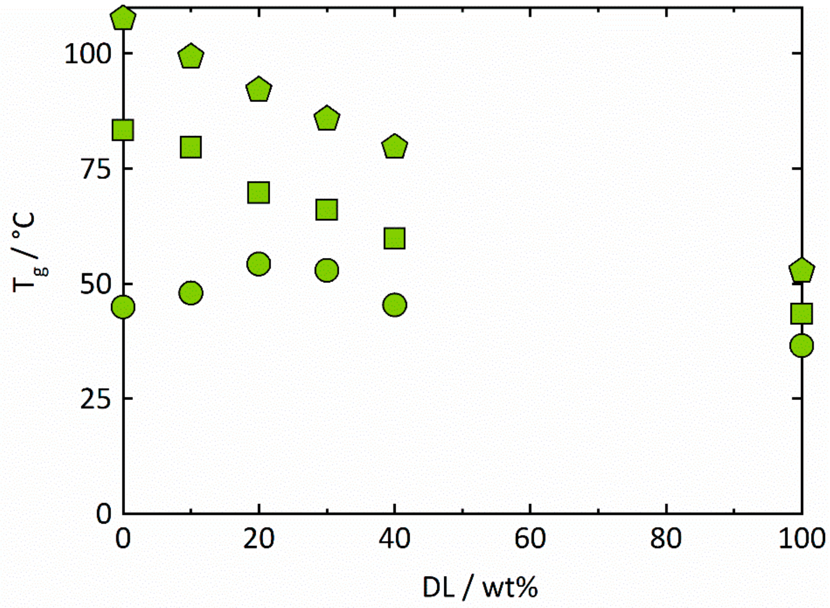

Figure S4. Glass-transition temperatures versus DL for RIT/PVPVA ASDs measured in this work after storage at 25 °C and RHs of 0% (pentagons), 23% (squares), and 53% (circles).

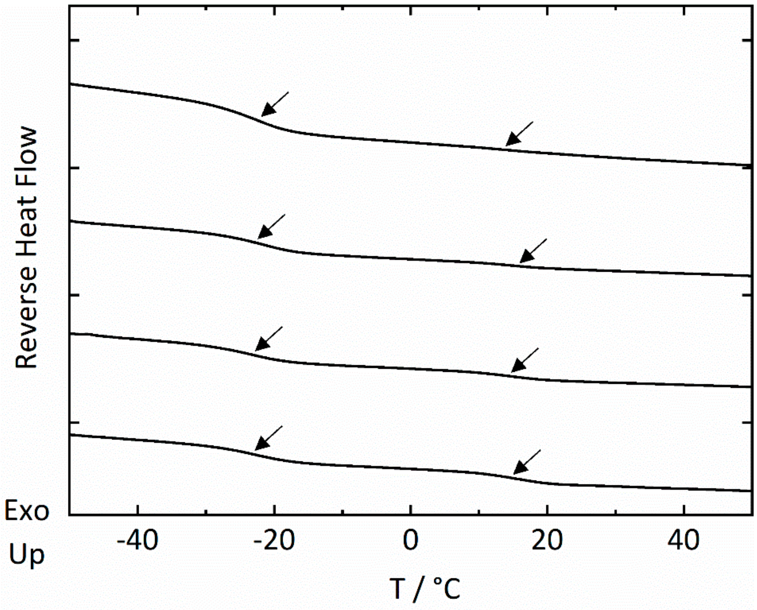

Figure S5. mDSC scans of RIT/PVPVA ASDs measured in this work after storage at 25 °C and 94% RH for DLs of 10wt%, 20wt%, 30wt%, and 40wt% from top to bottom. Arrows indicate glass-transition temperatures.

Author Contributions: Conceptualization, A.K., A.D., K.V., S.D., S.O.K., M.D., and G.S.; methodology, A.K., A.D., K.V., S.D., S.O.K., M.D., and G.S.; software, A.K. and A.D.; validation, A.K., A.D., K.V., S.D., S.O.K., M.D., and G.S.; formal analysis, A.K.; investigation, A.K.; resources, M.D. and G.S.; data curation, A.K. and A.D.; writing—original draft preparation, A.K.; writing—review and editing, A.K., A.D., K.V., S.D., S.O.K., M.D., and G.S.; visualization, A.K.; supervision, A.D., K.V., S.D., S.O.K., M.D., and G.S.; project administration, A.D., K.V., S.D., S.O.K., M.D., and G.S.; funding acquisition, M.D. and G.S. All authors have read and agreed to the published version of the manuscript.

Funding: This study was funded by AbbVie.

Institutional Review Board Statement: Not applicable.

Informed Consent Statement: Not applicable.

Data Availability Statement: Data is contained within the Article or Supplementary Material.

Acknowledgements: The authors thank Katharina Clemens for contributing to this work with mDSC measurements.

Conflicts of Interest: This study was funded by AbbVie. AbbVie participated in the study design, research, data collection, analysis, and interpretation of data, as well as writing, reviewing, and approving the publication. A.K., K.V., S.D., S.O.K., and M.D. are AbbVie employees and may own AbbVie stock/options. G.S. is a professor, A.D. is a postdoc, and A.K. is a Ph.D. student at the Department of Biochemical and Chemical Engineering at TU Dortmund University, and they have no conflict of interest to report.

Abbreviations

API, active pharmaceutical ingredient; ASD, amorphous solid dispersion; DL, drug load; LCST, lower critical solution temperature; mDSC, modulated differential scanning calorimetry; PET, polyethylene terephthalate; PC-SAFT, Perturbed-Chain Statistical Associating Fluid Theory; PVPVA, poly(vinylpyrrolidone-co-vinyl acetate); RH, relative humidity; RIT, ritonavir; UCST, upper critical solution temperature; VCM, vacuum compression molding.

References

1. Xi, H.; Ren, J.; Novak, J.M.; Kemp, E.; Johnson, G.; Klinzing, G.; Johnson, M.A.; Xu, W. The Effect of Inorganic Salt on Disintegration of Tablets with High Loading of Amorphous Solid Dispersion Containing Copovidone. *Pharm. Res.* **2020**, *37*, 70. https://doi.org/10.1007/s11095-020-2772-7.
2. Ritters, L.; Reichl, S. Influence of amorphous solid Dispersions of Paracetamol and PVP VA 64 on the thermoresponsive Behaviour of the Polymer. In Proceedings of the SPhERe Proceedings: 3rd International Symposium on Pharmaceutical Engineering Research, Braunschweig, Germany, 25–27 September 2019. https://doi.org/10.24355/DBBS.084-202001221403-0.
3. Taylor, L.S.; Langkilde, F.W.; Zografi, G. Fourier transform Raman spectroscopic study of the interaction of water vapor with amorphous polymers. *J. Pharm. Sci.* **2001**, *90*, 888–901. https://doi.org/10.1002/jps.1041.
4. Ilevbare, G.A.; Taylor, L.S. Liquid–Liquid Phase Separation in Highly Supersaturated Aqueous Solutions of Poorly Water-Soluble Drugs: Implications for Solubility Enhancing Formulations. *Cryst. Growth Des.* **2013**, *13*, 1497–1509. https://doi.org/10.1021/cg301679h.
5. Sun, Y.; Deac, A.; Zhang, G.G.Z. Assessing Physical Stability of Colloidal Dispersions Using a Turbiscan Optical Analyzer. *Mol. Pharm.* **2019**, *16*, 877–885. https://doi.org/10.1021/acs.molpharmaceut.8b01194.
6. Dohrn, S.; Luebbert, C.; Lehmkemper, K.; Kyeremateng, S.O.; Degenhardt, M.; Sadowski, G. Solvent influence on the phase behavior and glass transition of Amorphous Solid Dispersions. *Eur. J. Pharm. Biopharm.* **2021**, *158*, 132–142. https://doi.org/10.1016/j.ejpb.2020.11.002.
